# Anxiety and Mood Disruption in Collegiate Athletes Acutely Following Mild Traumatic Brain Injury

**DOI:** 10.3390/diagnostics14121276

**Published:** 2024-06-17

**Authors:** Rachel Zhang, Michael Martyna, Jordan Cornwell, Masaru Teramoto, Mollie Selfridge, Amanda Brown, Jamshid Ghajar, Angela Lumba-Brown

**Affiliations:** 1 Department of Emergency Medicine, Stanford University, Stanford, CA 94304, USA; 2 Department of Psychiatry, Stanford University, Stanford, CA 94304, USA; mrmartyn@ualberta.ca; 3 Department of Physical Medicine & Rehabilitation, University of Utah, Salt Lake City, UT 84108, USA; 4 Department of Sports Medicine, Stanford University, Stanford, CA 94304, USA; mselfridge@stanford.edu (M.S.);; 5 Department of Neurosurgery, Brain Performance Center, Stanford University, Stanford, CA 94304, USA

**Keywords:** mild traumatic brain injury, sports-related concussion, mood and anxiety symptoms, post-concussion symptoms, head injury

## Abstract

Objective: To report the symptom burden of anxiety and mood-related indicators following mTBI in collegiate student-athletes. Study Design: Retrospective cohort study of varsity collegiate athletes. Setting: University sports medicine at a tertiary care center. Patients: Division I college varsity athletes diagnosed with mTBI at a single institution between 2016 and 2019. Independent Variables: Pre- and post-injury. Main Outcome Measures: Comparisons between baseline testing and post-mTBI symptom scale assessments were made to determine changes in scores at the individual and group levels. The primary outcome was the prevalence of post-mTBI symptoms from within 72 h of injury through return to play. Associations with sport, sex, age, and return-to-play time were included. Results: Compared to baseline, mood and anxiety symptom scores were significantly higher acutely following mTBI (2.1 ± 3.3 vs. 14.3 ± 12.2; *p* < 0.001). A family history of migraine was significantly associated with higher mood and anxiety symptom scores (20.0 ± 14.9 with history vs. 13.3 ± 11.3 without history; *p* = 0.042). Mood and anxiety symptom scores were highly correlated with non-mood and anxiety symptom scores for all athletes, including the subgroup with prolonged symptoms (r = 0.769; *p* < 0.001). Conclusions: Symptoms of anxiety or mood disruption are common during the acute period post-injury in varsity college athletes. Risk factors for higher symptom reports immediately following mTBI and for prolonged symptoms (>10 days) included female sex, those with a family history of migraine, and those with an overall higher symptom burden post-injury.

## 1. Introduction

Anxiety and mood disruption occur acutely following mild traumatic brain injury (mTBI) and are often misidentified, not adequately assessed, and not discussed with patients [1,2,3]. MTBI is a disruption in the normal functioning of the brain following head injury and is associated with five common phenotypic profiles or subtypes: anxiety and mood disruption, vestibular impairment, cognitive impairment, headache or migraine, and oculomotor impairment [4,5,6]. In 2015, approximately 42 million people worldwide reportedly had mTBI [7,8]. Anxiety and mood disruption are common following mTBI; patients experience delays in care and a decrease in their quality of life [9,10]. The association between mTBI and mental health, represented by self-reports of anxiety and mood disruption in the acute and sub-acute time periods following injury, remains largely unexplored, even though there is an indication of a strong connection [11]. Varsity student-athletes sustaining mTBI represent a particularly vulnerable population to the effects of anxiety and mood disruption as they are often away from family support and are balancing rigorous academics with competitive athletics, resulting in significant stress [12,13]. For example, student-athletes dealing with mTBIs have more pronounced maladaptive coping compared to student-athletes with orthopedic injuries [14]. One study found children hospitalized for mTBIs were nine times more likely to have mood and anxiety disruption compared to those with orthopedic injuries [14,15]. In addition, children with mTBIs were four times more likely to have a new anxiety diagnosis compared to orthopedic controls [14]. Multiple studies explore the psychological effects of mTBI; however, these are not well described [16,17,18]. More recently, emotion recognition has become prevalent in affective computing [17]. However, this is still in its early stages. One particular research project proposed SST-Net, which is a 3D dense network with meta-learning [17]. This type of programming can eventually be included in mood and anxiety symptom evaluation.

Post-mTBI symptoms, including mood and anxiety, have been reported to remain elevated in 12% of one sample of individuals 12 months after injury [16]. This may be due to the fact that routine activities and social contact are often disrupted as part of post-mTBI symptom management [16]. Post-mTBI depressive symptoms are known to predict other symptoms, which indicates that life disruptions can result in a lower mood [16]. Social contact and support post-mTBI have been shown to improve quality of life and return to physical health [16].

Mild traumatic brain injury can result in brain changes, leading to various changes seen in individuals following injury. A complicating factor when evaluating anxiety and mood disruption symptoms is the overlap between traditional TBI symptoms and those associated with mental health conditions [15]. Examples include how impaired concentration as a cognitive deficit exists in the criteria of major depressive disorder, as well as the vestibular symptoms following mTBI also being part of the criteria of panic attacks [15]. Evidence has shown that metabolic disturbances seen post-TBI can be similar to those in patients diagnosed with clinical depression [15].

Pre-morbid, event-related, and post-morbid factors may contribute to psychological changes following a head injury. Risk factors such as mental health history and biological sex have been previously described [15]. Mental health history includes the diagnosed condition and prior treatment. Mental health history is a risk factor for greater overall mTBI symptoms as well as increased post-injury mood and anxiety disruption [15]. In addition, research has shown that individuals with a mental health history are at a higher risk of developing a new mental health condition [15]. This is found to somewhat include individuals with a family history of mental health conditions as they are predisposed to developing a condition [15]. Athletes identified as high-risk can be monitored more closely and prescribed a focused behavioral treatment approach [15]. Furthermore, females may report more symptoms than their male counterparts [15]. However, articles acknowledge that mood and anxiety disorders are more prevalent among females in the general public [15]. Other reasons include the existence of biological factors and different cultures between males and females [15].

As for event-related factors, brain changes due to mTBIs have been reported. These symptom burdens may result from common neurochemical disturbances that exist owing to both mTBI and mood and anxiety disruption. There is evidence of serotonin disruption and decreased dopamine levels in the prefrontal cortex and brainstem of patients with mTBI [15]. Post-mTBI symptoms affect a patient’s daily functioning, similar to the effects of mood and anxiety disruption [15].

Post-morbid factors include treatment methods and recovery trajectories. A patient’s response to injury, including coping style or emotional regulation, represents another factor in anxiety and mood disruption following mTBI [15]. The coping style post-mTBI is extremely important when evaluating anxiety and mood disruption. There are reports that those with an avoidant/passive coping strategy tend to have higher reports of mood disruption [15]. An individual’s ability to emotionally regulate themselves also affects their post-TBI symptoms. Studies have found that individuals with higher resilience demonstrate less mood and anxiety disruption [15]. Possible considerations include evaluating how athletes have responded to prior injuries or adverse events to better understand their ability to tolerate distress [15]. Interestingly, when measuring social competence in children with TBIs of different severities, those with mTBIs displayed the poorest social competence [16]. Individuals with mTBI may receive less support than those with more severe injuries [16]. mTBIs are often referred to as “invisible injuries” [16]. A longitudinal study followed children with a history of mTBI throughout their childhood. These children were at greater risk of attention-deficit/hyperactivity, conduct, or oppositional defiant disorder [16]. This was significantly different from that observed in children who experienced orthopedic injuries [16].

Common post-mTBI symptoms include headache, dizziness, sensory sensitivity, difficulty concentrating, and sleep disturbances, all of which can negatively affect daily life [16]. Sleep allows the brain to recover. However, excessive sleep can be detrimental. Many individuals report excessive sleep or difficulty falling asleep post-mTBI [16]. Regarding headaches, the literature notes the difficulty in distinguishing between post-mTBI headache pain and chronic pain [16]. These all require a balancing act and careful monitoring to optimize recovery. Up to 73% of athletes with mTBI may need academic accommodation [16]. However, one study found that only 53% of school nurses and 44% of athletic trainers were aware of the existing guidelines for returning-to-school policies and support systems [16]. Mismanagement of recovery can be detrimental for student-athletes as they manage emotional distress and academic struggles. One study reported that forty-five percent of 170 students believed they returned to school too soon, and this negatively affected their symptoms [16]. However, this is a balancing act, as those with moderate levels of mental and physical activity performed better in the weeks following mTBI [16]. Every individual’s recovery can be different. Further analysis of the manifestation of symptoms and the importance of symptom monitoring is crucial [16].

This work aimed to quantify symptoms of anxiety and mood disruption in the acute and sub-acute time periods following mTBI by examining self-reported symptoms using the commonly used post-mTBI symptom rating tool—the Sports Concussion Assessment Tool 5 (SCAT-5).

## 2. Methods

### 2.1. Post-mTBI Symptom Scale

The 22-item symptom scale in the SCAT-5 is based on the Acute Concussion Evaluation (ACE). The ACE has established construct and moderate subscale validity, including the emotionality subscale (Gioia, Gerard, Collins, Michael & Isquith, Peter. (2008). Improving Identification and Diagnosis of Mild Traumatic Brain Injury With Evidence: Psychometric Support for the Acute Concussion Evaluation. Journal of Head Trauma Rehabilitation, 23, 230–242. https://doi.org/10.1097/01.HTR.0000327255.38881.ca). The SCAT-5 includes “immediate on-field assessment, cervical spine assessment, neurological screening, Maddocks Questions for Memory Assessment, the Glasgow Coma Scale (GSC), a symptom evaluation, the Standardized Assessment of Concussion (SAC), and the modified Balance Error Scoring System (mBESS) [19]”.

In this study, the emotionality subscore questions were used as the primary depression and anxiety symptoms. The associated depression and anxiety symptoms are derivatives of the *Diagnostic and statistical manual of mental disorders* (5th ed.) (DSM-5) criteria for diagnosing major depressive disorder and generalized anxiety disorder (American Psychiatric Association. (2013). *Diagnostic and statistical manual of mental disorders* (5th ed.).

Symptoms were divided into two groups: mood and anxiety versus non-mood and anxiety. Primary mood and anxiety symptoms included sadness, nervousness/anxiousness, and feeling more emotional. Associated symptoms included drowsiness, trouble falling asleep, fatigue, difficulty concentrating, not “feeling right”, “feeling in a fog”, and feeling more slowed down than usual. The non-mood and anxiety symptoms included headache, pressure in the head, neck pain, nausea/vomiting, dizziness, blurred vision, balance problems, sensitivity to light, and sensitivity to noise.

### 2.2. Data Analysis

Descriptive statistics were calculated for athletes’ demographics, sport, repeat mTBIs, chronic health conditions (including depression), and family history of migraine. Specifically, mean and standard deviation (SD) were used for continuous variables, whereas categorical variables were summarized with frequencies and percentages. A preliminary examination of the data revealed that the symptom scores were positively skewed. Transformations did not yield normally distributed or symmetrical data. Hence, nonparametric tests were performed to determine the significance of the symptom scores. In particular, the Wilcoxon matched-pairs signed-rank test was used to compare symptom scores between baseline and post-injury separately for all injury cases and those excluding repeat instances. Further, post-injury symptom scores were compared by demographics, type of sport, repeat instances, depression, and family history of migraine. Specifically, two-group and three-group comparisons were made using the Wilcoxon–Mann–Whitney test and Kruskal–Wallis equality-of-populations rank test, respectively. The Pearson correlation coefficient (*r*) was calculated to examine the association between post-injury symptom scores and age. If any of these variables were significant, they were entered into a linear regression model as covariates, along with the baseline symptom score as another covariate, while calculating bootstrap standard errors with 1000 replications to account for non-normally-distributed post-injury symptom scores. These analyses were repeated for athletes with prolonged symptoms, which was defined as the last SCAT-5 date greater than 10 days [20]. In addition, symptom scores were compared between athletes with prolonged symptoms and those with no prolonged symptoms using the Wilcoxon–Mann–Whitney test. Lastly, *r* was calculated and examined to compare the post-injury-associated symptom scores with the post-injury primary symptom scores. All analyses were performed using Stata/MP 17.0 (StataCorp LLC, College Station, TX, USA).

### 2.3. Results

A total of 129 discrete mTBIs were analyzed in this study, including 33 injuries with prolonged symptoms (i.e., >10 days since the last SCAT-5 date) [20]. Demographic variables are summarized in Table 1. Approximately 60% of mTBIs occurred in male athletes (59.7%; *n* = 77), and almost 30% of mTBIs occurred in football players (29.5%; *n* = 38). Repeated mTBIs in a single individual consisted of 18.6% (*n* = 24) and 5.4% (*n* = 7) of the data at 392 ± 308 days and 227 ± 234 days since the previous injury, respectively.

**Figure 1:**
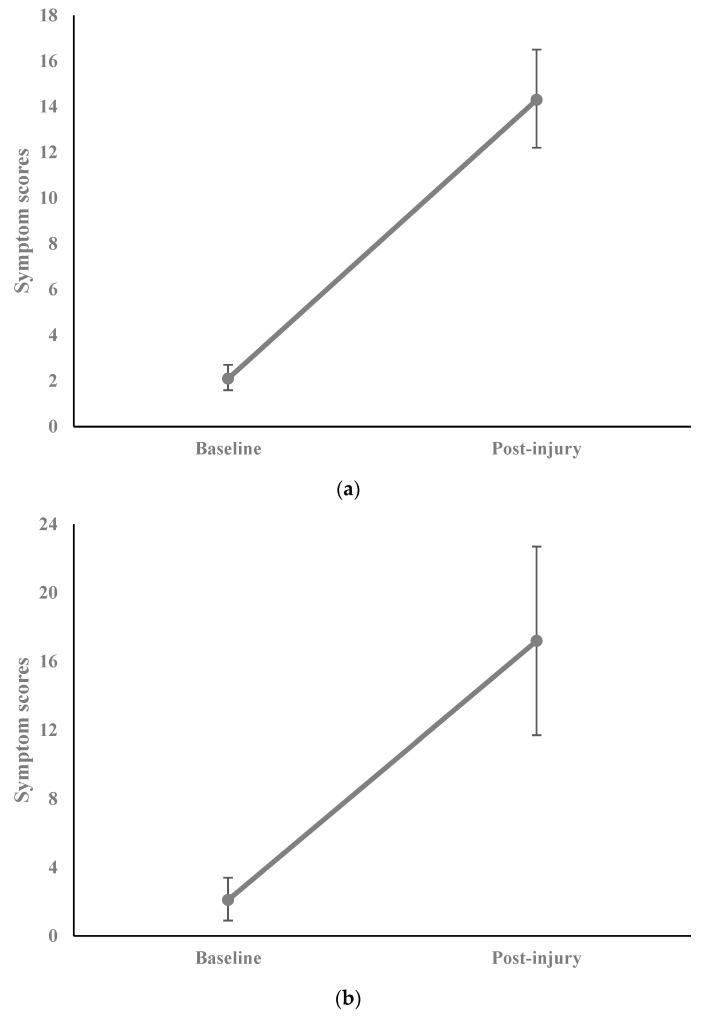
(**a**) Symptom scores at baseline and post-injury. (**b**) Symptom scores at baseline and post-injury for athletes with prolonged symptoms.

Symptom scores were significantly higher after injury than at baseline (*n* = 129; 14.3 ± 12.2 vs. 2.1 ± 3.3; difference = 12.2 ± 11.9; *p* < 0.001; Figure 1a). This finding held true even after excluding repeat instances (*n* = 98; 14.5 ± 12.6 vs. 2.4 ± 3.5; difference = 12.1 ± 12.3; *p* < 0.001). Among the covariates, a medical history of depression and a family history of migraine were significantly associated with higher post-injury symptom scores (*p* = 0.049 and 0.042, respectively; Table 2. According to the linear regression model with bootstrap standard errors, after adjusting for baseline symptom scores, a family history of migraine, but not depression, was still significantly associated with higher post-injury symptom scores (*p* = 0.041). Specifically, athletes with a family history of migraine reported an approximately 6.5-point-higher symptom burden on symptom scores than those with no such history.

In terms of discrete mTBIs in which patients exhibited prolonged symptoms (i.e., >10 days since last SCAT-5 date), symptom scores were significantly higher after injury than at baseline (*n* = 33; 17.2 ± 15.7 vs. 2.1 ± 3.7; difference = 15.1 ± 14.7; *p* < 0.001; Figure 1b), with a similar finding for injuries excluding repeat instances (*n* = 26; 18.8 ± 16.2 vs. 2.5 ± 4.1; difference = 16.3 ± 15.3; *p* < 0.001). Sex and contact sport were significantly associated with post-injury symptom scores (*p* = 0.008 and 0.012, respectively; Table 3). The linear regression model with bootstrap standard errors revealed that, after adjusting for baseline symptom scores, neither sex nor contact sport were significantly associated with post-injury symptom scores (*p* > 0.05).

**Figure 2:**
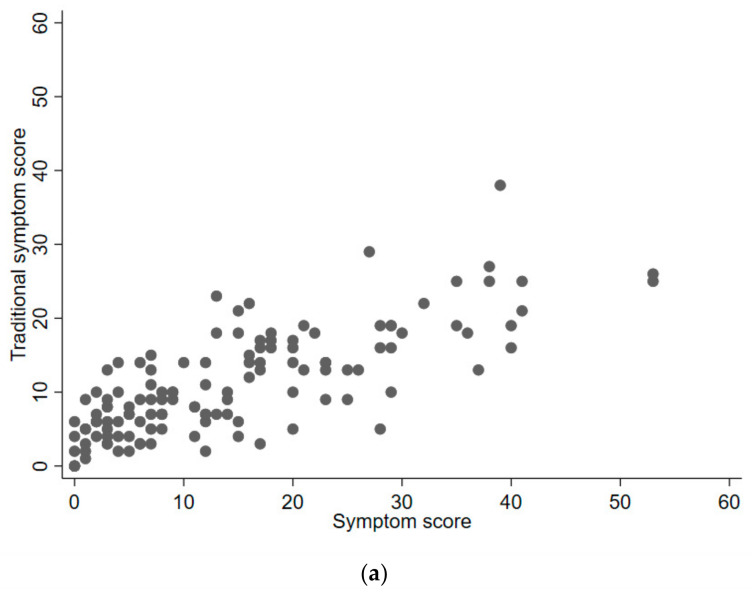

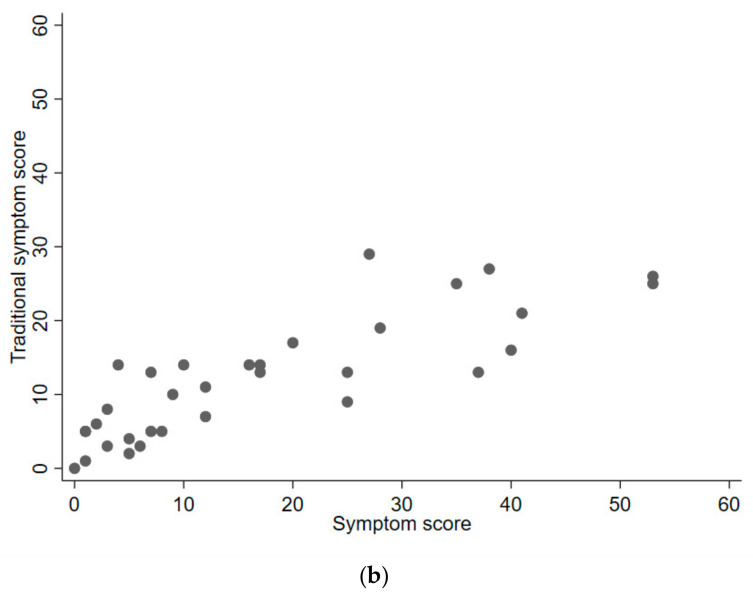
(**a**) Scatterplot of symptom scores and traditional symptom scores. (**b**) Scatterplot of symptom scores and traditional symptom scores in athletes with prolonged symptoms.

There was no significant difference in post-injury symptom scores between athletes with prolonged symptoms and those with no prolonged symptoms (17.2 ± 15.7 vs. 13.4 ± 10.6; *p* = 0.501). Post-injury symptom scores were significantly correlated with post-injury traditional scores among all injuries, as well as with prolonged symptoms (*p* < 0.001; Figure 2a,b).

## 3. Discussion

The categories of sex, family history of migraine, depression, and contact/non-contact sport all appeared to be significant factors in determining mood and anxiety symptom burden post-injury in this study. As stated by previous research, athletes recovering from mTBI are more likely to have depression symptoms compared to healthy controls [21]. There is a prospective study that details a prediction model for major depressive episodes following mTBI [22]. Within three months post-injury, 17% of patients with mTBI developed a major depressive episode [22]. This number increased to 33% within the first year after injury [22]. A retrospective study found that the lifetime risk of a major depressive episode was higher in patients with mTBI than in those with an extracranial injury [22].

One study identified four neurobehavioral phenotypes in patients following TBI: emotionally resilient, cognitively impaired, cognitively resilient, and neuropsychiatrically distressed [23]. In the study, these phenotypes predicted 6-month outcomes better than the traditional chart review and injury severity measures. These findings highlight the significance of identifying risk factors or high-risk phenotypes, as this can lead to improved targeting of appropriate interventions. In a heterogeneous population, such as mTBI, this could be of the utmost importance. In their study, as in ours, female sex was associated with phenotypes that had poorer outcomes. Another study found that females had greater aggregate symptom severity post-mTBI and took longer to return to baseline [24]. However, it is important to note that females have a greater severity of symptoms at baseline as well [24]. This has been well documented in the literature. These significant findings are echoed in our work, as females had significantly higher mood and anxiety symptom scores, including those with prolonged symptoms.

Our finding of no significant association between a history of depression and poorer outcomes is unlike that of previous studies. However, although our study only included five participants with a history of depression, all subjects reported at least one symptom of anxiety or mood disruption during the acute period post-injury. We observed a similar trend toward a history of depression being related to more and longer symptom burden. Athletes are often commended for their resilience; however, cessation of their sport can be especially challenging. Being pulled away from sports due to injury threatens the identity that the individual has built [25]. A previous study found that resilience was positively correlated with mental health. More importantly, there was a weakly positive correlation between athletic identity and anxiety and loneliness [25]. A three-factor model has been developed to better understand athletic identity [25]. This includes social identity, exclusivity, and negative affectivity [25]. Social identity represents how much of an individual’s identity is being an athlete, exclusivity considers how much self-worth is dependent on athletic identity, and negative affectivity describes the negative emotions of being away from the sport and not participating [25]. The results of Knowles et al. support our findings that a history of depression likely further negatively exacerbates people’s psychological state. There is a growing body of evidence that elite athletes are not as resilient to mental health issues as was initially assumed. College is already an at-risk period for the development of depression symptoms [26]. A 2014 nationwide survey by the American College Health Association found that over 30% of college students reported some degree of feeling depressed over the past 12 months [27]. In addition to these risk factors, college athletes have to meet the physical and psychological demands of their sport, which increases their risk [26]. There is no question regarding the physical and mental benefits of sports participation; however, its protective benefits have been overestimated. According to the NIH, the prevalence of a major depressive episode in 2019 was highest in individuals between the ages of eighteen and twenty-five. Wolanin et al. report that the clinically relevant depressive symptom prevalence rate among 465 college athletes was at a striking 23.7% [27]. The prevalence of a moderate-to-severe level was 6.3%. A more recent study found that out of 950 NCAA Division I athletes, 33.2% experienced some symptoms of depression [26].

The finding that a history of depression is not significantly related to mTBI outcomes is distinct from the adult mTBI literature. Our study had a low proportion of participants with a history of depression at 4% (*n* = 5), whereas a general population of adults would be expected to be nearer to 10% (DSM-5). This low percentage is supported by a previous study that found that athletes scored higher in the mental toughness category [28]. Mental toughness is known to be linked to developmental experiences, and sports participation offers challenges and commitment [28]. In the scientific literature, mental toughness is evaluated through interviews and mental toughness inventories [28]. Mental toughness includes hope, optimism, perseverance, and resilience [28]. Furthermore, athletes might come from groups of cognitively and emotionally resilient phenotypes [23]. Another potential explanation for this difference in our specific population is that the collegiate athlete population may select those who have greater access to medical assistance and intrinsic social protective factors, such as being a member of a team and having a higher education level.

Our study adds to the literature by clarifying existing trends in mTBI recovery, as well as by contributing longitudinal quantitative data. A retrospective study by Martinez et al. found that cognitive impairments, visual exam findings, and vestibular exam findings all increased the odds of delayed recovery [29]. Our study builds upon these findings by showing that there is a high positive correlation between non-mood/anxiety symptom scores and mood/anxiety-related symptoms for all individuals, as well as for those showing prolonged symptoms. Our study shows that the overall symptom burden post-injury is associated with prolonged symptoms. Others have found that a history of prior mTBI is associated with a higher risk of mTBI symptoms greater than one week for both football and non-football players. Our study did not find the same, as individuals with prior mTBIs did not have significantly higher post-mTBI mood and anxiety scores.

Most subjects reported at least one symptom of anxiety or mood disruption during the acute period post-injury. Risk factors for higher mood and anxiety symptom scores immediately following mTBI and for prolonged symptoms (>10 days) included female sex, a family history of migraine, and an overall higher symptom burden post-injury, representing an important aspect of post-mTBI symptom assessment in acute settings and prognostic counseling. This study provides evidence of a high prevalence of anxiety and mood disruption following mTBI. Targeted rehabilitation and neuropsychological follow-up for mood- and anxiety-related symptoms may improve mTBI recovery.

A strength of this study is that the database utilized consisted of both baseline and post-injury data. The baseline data were collected pre-injury and were not a retrospective analysis of the premorbid state. The data were collected at several homogenous timepoints by trained clinicians. This allowed for reliable data collection and provided a longitudinal analysis for a more complete picture of any trends or changes. Using the well-established SCAT-5 evaluation only adds to the validity of these data. To our knowledge, this study is the first to examine mood and anxiety disruption in collegiate athletes following mTBI diagnosis.

A limitation of this study is the sample size. A total of 119 athletes and 130 mTBI patients were included in this study. Sixteen sports were included, of which multiple had less than three reported mTBIs, whereas the majority were derived from two sports. Some data were excluded due to missing information. As discussed, only five participants had a history of depression, which is a previously identified risk factor. Another limitation is the subjective measures of symptom evaluation. SCAT-5 is based on patient self-reports, although this is not unique to this study.

The next steps in the research include expanding both the sample size and length of follow-up to further elucidate the recovery trajectory of collegiate athletes following mTBI. This information could also inform intervention studies aimed at improving the outcomes of these athletes.

## 4. Conclusions

Symptoms of anxiety or mood disruption are common during the acute post-injury period in varsity college athletes. Risk factors for higher symptom reports immediately following mTBI and for prolonged symptoms (>10 days) included female sex, a family history of migraine, and overall higher symptom burden post-injury. Our research adds to the existing literature by providing longitudinal quantitative data as well as supporting existing mTBI recovery trends. This study indicates the importance of targeted rehabilitation for improving mTBI recovery.

## Figures and Tables

**Table 1:** Demographics of injured athletes (*N* = 129).

Variable	Frequency (%)
Sex	
Male	77 (59.7)
Female	52 (40.3)
Sport	
Baseball	2 (1.6)
Basketball	8 (6.2)
Beach Volleyball	4 (3.1)
Bicycle Accident	5 (3.9)
Fencing	1 (0.8)
Field Hockey	2 (1.6)
Football	38 (29.5)
Gymnastics	4 (3.1)
Lacrosse	6 (4.7)
Other	2 (1.6)
Sailing	3 (2.3)
Soccer	4 (3.1)
Softball	4 (3.1)
Swimming and Diving	3 (2.3)
Track and Field	1 (0.8)
Volleyball	5 (3.9)
Water Polo	8 (6.2)
Wrestling	16 (12.4)
No data	13 (10.1)
Repeat instances	
One	98 (76.0)
Two *	24 (18.6)
Three **	7 (5.4)
Prolonged symptoms	
Yes	33 (25.6)
No	68 (52.7)
No data	28 (21.7)
Depression	
Yes	5 (3.9)
No	107 (82.9)
No data	17 (13.2)
Family history of migraine	
Yes	29 (22.5)
No	89 (69.0)
No data	11 (8.5)
Age [mean (SD)]	19.8 (1.4)
Baseline symptom score [mean (SD)]	2.1 (3.3)
Post-injury symptom score [mean (SD)]	14.3 (12.2)
Traditional symptom score [mean (SD)]	11.0 (7.1)

* 392 ± 308 days since previous (1st) injury. ** 227 ± 234 days since previous (2nd) injury.

**Table 2:** Post-injury symptom scores by covariate.

Grouping Variable	Post-Injury Symptom Score [Mean (SD)]	*p*
Sex		0.117 *
Male (*n* = 77)	12.6 (10.6)	
Female (*n* = 52)	16.9 (14.0)	
Sport		0.563 *
Football (*n* = 38)	13.1 (12.3)	
Non-football (*n* = 78)	14.1 (12.5)	
Contact sport		0.193 *
Contact (*n* = 74)	12.5 (11.7)	
Non-contact (*n* = 40)	15.8 (13.8)	
Repeat instances		0.898 **
One (*n* = 98)	14.5 (12.6)	
Two (*n* = 24)	14.3 (11.7)	
≥ Three (*n* = 7)	11.7 (10.0)	
Depression		0.049 *
Yes (*n* = 5)	26.2 (14.3)	
No (*n* = 107)	14.0 (12.3)	
Family history of migraine		0.042 *
Yes (*n* = 29)	20.0 (14.9)	
No (*n* = 89)	13.3 (11.3)	
Age (*n* = 128); [*r* (*p*)]	0.083 (0.355)	

*r* = Pearson’s correlation coefficient. * From the exact Wilcoxon–Mann–Whitney test. ** From the Kruskal–Wallis equality-of-populations rank test.

**Table 3:** Post-injury symptom scores by covariate for athletes with prolonged symptoms.

Grouping Variable	Post-Injury Symptom Score [Mean (SD)]	*p*
Sex		0.008 *
Male (*n* = 19)	10.6 (10.8)	
Female (*n* = 14)	26.2 (17.2)	
Sport		0.356 *
Football (*n* = 10)	12.3 (13.2)	
Non-football (*n* = 20)	18.7 (17.1)	
Contact sport		0.012 *
Contact (*n* = 20)	10.9 (12.2)	
Non-contact (*n* = 9)	29.0 (17.8)	
Repeat instances		0.130 **
One (*n* = 26)	18.8 (16.2)	
Two (*n* = 5)	15.2 (14.0)	
≥Three (*n* = 2)	1.5 (2.1)	
Depression		N/A
Yes (*n* = 0)	0.0 (0.0)	
No (*n* = 28)	18.2 (16.0)	
Family history of migraine		0.080 *
Yes (*n* = 10)	25.6 (16.5)	
No (*n* = 20)	14.8 (14.9)	
Age (*n* = 33); [*r* (*p*)]	−0.130 (0.471)	

*r* = Pearson’s correlation coefficient. * From exact Wilcoxon–Mann–Whitney test. ** From Kruskal–Wallis equality-of-populations rank test.

## Data Availability

The data presented in this study are available on request from the corresponding author due to patient confidentiality.
